# Supplementary material for: Differentiation alters stem cell nuclear architecture, mechanics, and mechano-sensitivity
Source: eLife. 2016 Nov 30;5:e18207. doi: 10.7554/eLife.18207 (PMC5148611; doi:10.7554/eLife.18207)
Supplement: Source code 1. — DOI: http://dx.doi.org/10.7554/eLife.18207.024 [file elife-18207-code1.zip › ApplyThresh.docx]

function [IThresh] = ApplyThresh(I,T)
% This function receives the image to be thresholded and the threshold
% value. It then produces a thresholded image.
% I: image to be thresholded
% T: threshold value

clear row column int S sizerow sizecolumn R C
[row,column,~] = find(I>T);
S = length(row);
[sizerow,sizecolumn] = size(I);
IThresh = zeros(sizerow,sizecolumn);
for i = 1:S
 R = row(i,1);
 C = column(i,1);
 IThresh(R,C) = 1;
end

Not enough input arguments.

Error in ApplyThresh (line 8)
[row,column,~] = find(I>T);

[*Published with MATLAB® R2015b*](http://www.mathworks.com/products/matlab)
