# Supplementary material for: Differentiation alters stem cell nuclear architecture, mechanics, and mechano-sensitivity
Source: eLife. 2016 Nov 30;5:e18207. doi: 10.7554/eLife.18207 (PMC5148611; doi:10.7554/eLife.18207)
Supplement: Source code 1. — DOI: http://dx.doi.org/10.7554/eLife.18207.024 [file elife-18207-code1.zip › ExtractImage.docx]

function [IExtract] = ExtractImage(I,In)
% This function receives the image to be extracted to a black background
% and the thresholded image (having the pixels locating where the target
% is). This function then produces a target image with black background.
% I: target image
% In: thresholded image

clear row column int S sizerow sizecolumn R C
[row,column,~] = find(In>0);
S = length(row);
[sizerow,sizecolumn] = size(In);
IExtract = zeros(sizerow,sizecolumn);
for i = 1:S
 R = row(i,1);
 C = column(i,1);
 IExtract(R,C) = I(R,C);
end

Not enough input arguments.

Error in ExtractImage (line 9)
[row,column,~] = find(In>0);

[*Published with MATLAB® R2015b*](http://www.mathworks.com/products/matlab)
