# Supplementary material for: Differentiation alters stem cell nuclear architecture, mechanics, and mechano-sensitivity
Source: eLife. 2016 Nov 30;5:e18207. doi: 10.7554/eLife.18207 (PMC5148611; doi:10.7554/eLife.18207)
Supplement: Source code 1. — DOI: http://dx.doi.org/10.7554/eLife.18207.024 [file elife-18207-code1.zip › GeneratingPrint.docx]

function [PrintList] = GeneratingPrint(PrintIndex,q,PrintNameList,I2,I3,I4,I5,I6,I7,A1,I8,I9,I10,I11,I12,I13)
% This function receives an index describing the image matrices to save and
% the q-th image being processed.
% This function then produces .tif files of the chosen analysis matrices
% PrintIndex: The index of chosen matrices (m x 1)
% q: The q-th image being processed

Index = find(PrintIndex>0);
Cond = isempty(Index);
if Cond == 0
 for i = 1:size(Index,1)
 ImageChosen = Index(i,1);
 if ImageChosen == 1
 imwrite(I2,PrintNameList(q,i).name,'tif');
 elseif ImageChosen == 2
 imwrite(I3,PrintNameList(q,i).name,'tif');
 elseif ImageChosen == 3
 imwrite(I4,PrintNameList(q,i).name,'tif');
 elseif ImageChosen == 4
 imwrite(I5,PrintNameList(q,i).name,'tif');
 elseif ImageChosen == 5
 imwrite(I6,PrintNameList(q,i).name,'tif');
 elseif ImageChosen == 6
 imwrite(I7,PrintNameList(q,i).name,'tif');
 elseif ImageChosen == 7
 imwrite(A1,PrintNameList(q,i).name,'tif');
 elseif ImageChosen == 8
 imwrite(I8,PrintNameList(q,i).name,'tif');
 elseif ImageChosen == 9
 imwrite(I9,PrintNameList(q,i).name,'tif');
 elseif ImageChosen == 10
 imwrite(I10,PrintNameList(q,i).name,'tif');
 elseif ImageChosen == 11
 imwrite(I11,PrintNameList(q,i).name,'tif');
 elseif ImageChosen == 12
 imwrite(I12,PrintNameList(q,i).name,'tif');
 elseif ImageChosen == 13
 imwrite(I13,PrintNameList(q,i).name,'tif');
 end
 end
 PrintList = size(Index,1);
else
 PrintList = 0;
end

Not enough input arguments.

Error in GeneratingPrint (line 8)
Index = find(PrintIndex>0);

[*Published with MATLAB® R2015b*](http://www.mathworks.com/products/matlab)
