# Supplementary material for: Differentiation alters stem cell nuclear architecture, mechanics, and mechano-sensitivity
Source: eLife. 2016 Nov 30;5:e18207. doi: 10.7554/eLife.18207 (PMC5148611; doi:10.7554/eLife.18207)
Supplement: Source code 1. — DOI: http://dx.doi.org/10.7554/eLife.18207.024 [file elife-18207-code1.zip › GeneratingPrintName.docx]

function [PrintNameList] = GeneratingPrintName(PrintIndex,s)
% This function receives an Index, which indicates the chosen image
% matrices, and the number of images being processed.
% This function then produces the list of names of the chosen matrices
% PrintIndex: The index of chosen matrices (m x 1)
% s: The number of images being processed (1 x 1)

Index = find(PrintIndex>0);
Cond = isempty(Index);
if Cond == 0
 for i = 1:size(Index,1)
 ImageChosen = Index(i,1);
 if ImageChosen == 1
 PName = 'I2';
 elseif ImageChosen == 2
 PName = 'I3';
 elseif ImageChosen == 3
 PName = 'I4';
 elseif ImageChosen == 4
 PName = 'I5';
 elseif ImageChosen == 5
 PName = 'I6';
 elseif ImageChosen == 6
 PName = 'I7';
 elseif ImageChosen == 7
 PName = 'A1';
 elseif ImageChosen == 8
 PName = 'I8';
 elseif ImageChosen == 9
 PName = 'I9';
 elseif ImageChosen == 10
 PName = 'I10';
 elseif ImageChosen == 11
 PName = 'I11';
 elseif ImageChosen == 12
 PName = 'I12';
 elseif ImageChosen == 13
 PName = 'I13';
 end
 for j = 1:s
 PrintName = sprintf('%s-%03d.tif',PName,j);
 PrintNameList(j,i).name = PrintName;
 end
 end
else
 PrintNameList = 'NO PRINT CHOSEN';
end

Not enough input arguments.

Error in GeneratingPrintName (line 8)
Index = find(PrintIndex>0);

[*Published with MATLAB® R2015b*](http://www.mathworks.com/products/matlab)
