# Supplementary material for: Differentiation alters stem cell nuclear architecture, mechanics, and mechano-sensitivity
Source: eLife. 2016 Nov 30;5:e18207. doi: 10.7554/eLife.18207 (PMC5148611; doi:10.7554/eLife.18207)
Supplement: Source code 1. — DOI: http://dx.doi.org/10.7554/eLife.18207.024 [file elife-18207-code1.zip › Main_SobelNucleusAnalysisV1.docx]

# Chromatin condensation analysis using the sobel edge finding method.

Used in Heo et al. 2016. Author: Stephen D. Thorpe Institute: School of Engineering and Materials Science, Queen Mary University of London, London, UK.

ABOUT 1

VARIABLE DETAILS 1

IMAGE ANALYSIS STEP OUTPUT 1

INPUT VARIABLES 2

RESULTS OUTPUT SETUP 2

CORE ALGORITHM 2

RESULTS OUTPUT 4

## ABOUT

- These are the required inputs for the algorithm.
- Image files must be saved as 8 bit .tif files in either RGB or grey values.
- Each image must only contain 1 nuclei on a black background.
- All images should be placed in the working directory with the Matlab file and associated functions. The code will cycle through all images in folder.
- Associated functions required: ThreshMode.m, ApplyThresh.m, ExtractImage.m, GeneratingPrintName.m, GeneratingPrint.m,
- This code has been optimised for 512x512 pixel confocal image slides taken through the central plane of a Hoechst 33342 or DAPI stained nucleus using a x40 or x63 oil objective with a zoom to approx 50nm x-y voxel size. However, it could be adjusted for other image types.

## VARIABLE DETAILS

- XLfilename: The name given to the EXCEL file to be produced, this will contain the area, edge count and edge density from each image.
- filenames: This will search the folder for the image type specified.
- PixRedFaxtor: Image reduction factor. Set to 4. Increase to 8 if using 1026x1026 images, or reduce if using smaller images. This affects the sensitivity of the edge finding algorithm and the magnitude of the final edge density value.
- SobelThresh: The threshold value for the SOBEL edge detection. This affects the sensitivity of the edge finding algorithm and can be optomised for your image type.

## IMAGE ANALYSIS STEP OUTPUT

- Images can be output and saved for each step of the procedure.
- If you want to print out an image matrix, use the value 1. If not required, use the value 0.

I = original image I2 = original image smoothed by mean filter (6x) I3 = image of pixels that above the threshold (T) I4 = image of pixels that above the threshold with the holes filled I5 = image of the thresholded nucleus in black background plus intensity redistributed I6 = downsampled I5 into 128x128 (by a factor of 4) I7 = SOBEL image (logical) A1 = SOBEL image (uint8) I8 = image of pixels that above the threshold from 128x128 image (I6) I9 = image of pixels that above the threshold from 128x128 image with the holes filled I10 = the inner part of the nucleus (Region of Interest or ROI) I11 = perimeter of the ROI I12 = image of the SOBEL edge within the ROI I13 = image of the SOBEL edge within the ROI (I12) plus the perimeter of previous ROI (I11)

## INPUT VARIABLES

Variables described above for editing.

clear %clear workspace
clc %clear command window

XLfilename = 'Results AREA EDGECOUNT EDGEDENSITY.xlsx';
filenames = dir('*.tif');

PrintI2 = 0;
PrintI3 = 0;
PrintI4 = 0;
PrintI5 = 1;
PrintI6 = 0;
PrintI7 = 0;
PrintA1 = 0;
PrintI8 = 0;
PrintI9 = 0;
PrintI10 = 0;
PrintI11 = 0;
PrintI12 = 1;
PrintI13 = 0;

PixRedFactor = 4;
SobelThresh = 0.12;

## RESULTS OUTPUT SETUP

Produce file names for analysis step image matrices to be saved

PrintIndex = [PrintI2;PrintI3;PrintI4;PrintI5;PrintI6;PrintI7;PrintA1;...
 PrintI8;PrintI9;PrintI10;PrintI11;PrintI12;PrintI13];
s = numel(filenames);
% Create a list of analysis step images to be saved
[PrintNameList] = GeneratingPrintName(PrintIndex,s);

% Set up results table
Resultlist = {'Pixel reduction factor',PixRedFactor,' ',' ';...
 'Sobel threshold',SobelThresh,' ',' ';...
 'Filename','Area [pix]','Edges [pix]','EdgeDen [%]'};
hl = 3; %headerlines in results list for output

Undefined function 'GeneratingPrintName' for input arguments of type 'double'.

Error in Main_SobelNucleusAnalysisV1 (line 88)
[PrintNameList] = GeneratingPrintName(PrintIndex,s);

## CORE ALGORITHM

Core algorithm which loops through all images in folder analysing each one by one, saving results to Resultlist for output at end.

for q = 1:numel(filenames)
 % Load image
 I = imread(filenames(q).name);
 Resultlist{q+hl,1} = filenames(q,1).name;

 if size(I,3) == 3 %Check if RGB image
 % If RGB image, take blue "3" channel. Change to "1" for red or "2"
 % for green.
 I = I(:,:,3);
 end
 % Acquire threshold value for I using ThreshMode function. This is used
 % to select nucleus from background.
 [T] = ThreshMode(I);

 % Image average smoothening by (i)th times. Set to 4 to smoothen out
 % noise.
 I2 = I;
 for i = 1:4
 h = fspecial('average');
 I2 = imfilter(I2,h);
 end

 % Thereshold application to I2 to select nucleus in smoothened image
 [I3] = ApplyThresh(I2,T);
 I3 = logical(I3);

 %Hole-filling algorithm to fill in any hole (below threshold)regions in
 %nucleus
 I4 = imfill(I3,'holes');

 % Extract the nucleus from the original image to a black background
 [I5] = ExtractImage(I,I4);
 I5 = uint8(I5);

 % Intensity redistribution for I5
 A = max(max(I5));
 B = double(I5);
 C = double(A);
 I5 = (B/C)*255;
 I5 = uint8(I5);

 % Image reduction by a factor specified above (1/factor)
 PixRedFactor2 = 1/PixRedFactor;
 I6 = imresize(I5,PixRedFactor2);

 % Intensity redistribution for I6
 A = max(max(I6));
 B = double(I6);
 C = double(A);
 I6 = (B/C)*255;
 I6 = uint8(I6);

 % SOBEL edge detection application using threshold value specified above
 I7 = edge(I6,'sobel',SobelThresh);
 A1 = uint8(I7);
 A1 = A1*255;

 % Acquire threshold value for I6
 clear T;
 [T] = ThreshMode(I6);

 % Threshold application to I6
 [I8] = ApplyThresh(I6,T);
 I8 = logical(I8);

 % Hole-filling algorithm
 I9 = imfill(I8,'holes');

 % Perimeter subtraction by (n)th times as there will always be an edge
 % at the nucelus perimeter
 I10 = I9;
 n = 2;
 for i = 1:n
 I11 = bwperim(I10);
 I10 = I10-I11;
 I10 = logical(I10);
 end

 % Extract the SOBEL edges inside the nucleus onto a black background
 [I12] = ExtractImage(I7,I10);
 I12 = logical(I12);
 I13 = I12+I11;
 I13 = uint8(I13);
 I13 = I13*255;

 % Nucleus area
 [row,column,int] = find(I10>0);
 Area = length(row);
 Resultlist{q+hl,2} = Area;

 % Edge count
 edgecount = sum(sum(I12));
 Resultlist{q+hl,3} = edgecount;

 % Edge density (i.e. chromatin condensation parameter)
 edgeden = (edgecount/Area)*100;
 Resultlist{q+hl,4} = edgeden;

 I12 = uint8(I12);
 I12 = I12*255;

 % Export .tif files of each selected analysis step
 [PrintList] = GeneratingPrint(PrintIndex,q,PrintNameList,I2,I3,I4,...
 I5,I6,I7,A1,I8,I9,I10,I11,I12,I13);
end

## RESULTS OUTPUT

xlswrite(XLfilename,Resultlist);

[*Published with MATLAB® R2015b*](http://www.mathworks.com/products/matlab)
