# Supplementary material for: Differentiation alters stem cell nuclear architecture, mechanics, and mechano-sensitivity
Source: eLife. 2016 Nov 30;5:e18207. doi: 10.7554/eLife.18207 (PMC5148611; doi:10.7554/eLife.18207)
Supplement: Source code 1. — DOI: http://dx.doi.org/10.7554/eLife.18207.024 [file elife-18207-code1.zip › ThreshMode.docx]

function [Thresh] = ThreshMode(I)
% Function to apply threshold to 8 bit grey image by finding minimum in
% histogram of grey values.
I = double(I);
MaxInt = max(max(I));
MaxInt = single(MaxInt);

H = hist(I(:),0:MaxInt);

Iteration = 0;
Cond = 0;

while Cond == 0
 F = ones(1,3)/3;
 H = conv2(H,F,'same');

 Sy = size(H,2);
 Peak = 0;

 for i = 2:Sy-1
 if Peak < 3
 if H(i-1)<H(i) && H(i+1)<H(i)
 Peak = Peak+1;
 end
 end
 end

 if Peak > 2
 Cond = 0;
 else
 Cond = 1;
 end

 Iteration = Iteration + 1;

 if Iteration > 10000
 Thresh = 0;
 return
 end
end

for j = 2:MaxInt
 if H(j-1)>H(j) && H(j+1)>H(j)
 Thresh = j-1;
 end
end

Not enough input arguments.

Error in ThreshMode (line 4)
I = double(I);

[*Published with MATLAB® R2015b*](http://www.mathworks.com/products/matlab)
